# Metaphor Comprehension in Individuals with Autism Spectrum Disorder: Core Language Skills Matter

**DOI:** 10.1007/s10803-021-04922-z

**Published:** 2021-03-10

**Authors:** Tamara Kalandadze, Johan Braeken, Cecilia Brynskov, Kari-Anne Bottegaard Næss

**Affiliations:** 1 Department of Special Needs Education, University of Oslo, Oslo, Norway; 2 Present Address: Faculty of Education, Østfold University College, B R A Veien 4, P.O. 700, Halden, Norway; 3 CEMO: Centre for Educational Measurement, University of Oslo, Oslo, Norway; 4 Department of Nordic Studies and Linguistics, University of Copenhagen, Copenhagen, Denmark

**Keywords:** Autism, Figurative language, Metaphor, Pragmatics

## Abstract

Poor metaphor comprehension was considered a hallmark of autism spectrum disorder (ASD), but recent research has questioned the extent and the sources of these difficulties. In this cross-sectional study, we compared metaphor comprehension in individuals with ASD (*N* = 29) and individuals with typical development (TD; *N* = 31), and investigated the relationship between core language and metaphor comprehension. Individuals with ASD showed more difficulty but also a more variable performance in both metaphor and literal items of the task used than individuals with TD did. This indicates that core language ability accounts for metaphor comprehension and should be considered in future research and interventions aiming to improve metaphor comprehension in individuals with ASD.

**Supplementary Information:**

The online version contains supplementary material available at 10.1007/s10803-021-04922-z.

Autism spectrum disorder (ASD) is characterized by impaired social communication and stereotypical behaviours and interests (American Psychiatric Association, 2013). Individuals with ASD show considerable variability in their skills within structural aspects of language such as semantics and grammar. In contrast, difficulty within language pragmatics (i.e., the social use and understanding of language in contexts) is considered a hallmark feature of ASD (e.g., Lord & Paul, 1997; Tager-Flusberg & Joseph, 2003). Among difficulties within various pragmatic aspects, misinterpreting metaphors is considered universal in ASD (e.g., see Happé, 1993 for a pioneering study on metaphor comprehension in ASD).

Metaphor is a paradigmatic type of a figurative language in which there is a divergence between the encoded literal meaning of words and their occasion-specific use (Carston, 2017; Noveck et al., 2001). Metaphors are an essential part of oral and written language and communication (Bowdle & Gentner, 2005), and children and adolescents are frequently exposed to metaphors through conversations, education, literature, media communication, and films (e.g., Cameron, 2003; Colston & Kuiper, 2002; Golden, 2010; Katz, 2017; Nippold, 2016; Steen et al., 2010). Thus, not being able to understand metaphors can have a negative impact on daily life.

Although individuals with ASD have been shown to struggle with metaphor comprehension, neither the extent of difficulty within metaphor comprehension compared to individuals with TD nor the variables that can explain this difficulty has been investigated sufficiently so far. The aims of this study are (a) to investigate the extent of difficulties in metaphor comprehension in ASD compared to typical development (TD), and (b) to examine the potential relationships between metaphor comprehension and core language skills. The findings of this study will inform future research and practice in identifying targets for interventions customized for individuals with ASD.

## Metaphor Comprehension in ASD and in Typical Development (TD)

While children with TD are able to understand metaphors that are linguistically and cognitively age-appropriate (Pouscoulous, 2011), metaphor comprehension has been shown to be a great challenge even for verbally fluent individuals with ASD (Adachi et al., 2004; Vulchanova et al., 2015). This intriguing difference between these groups in metaphor comprehension has been investigated for more than three decades (see Kalandadze et al., 2019 for a review), and findings of older studies have contributed to the view that metaphor comprehension is universally impaired in ASD (Gernsbacher & Pripas-Kapit, 2012). However, more recent studies have not shown statistically significant differences in metaphor comprehension between individuals with ASD and individuals with TD (e.g., Gold et al., 2010; Hermann et al., 2013; Kasirer & Mashal, 2014; Mashal & Kasirer, 2011). Traditionally, compromised Theory of Mind (ToM) ability, that is, the ability to understand the mental states of others (Baron-Cohen et al., 1985) has been suggested as the main explanation of difficulties in metaphor comprehension (e.g., Happé, 1993). However, some of the subsequent studies proposed that difficulties in metaphor comprehension individuals with ASD often show, cannot be explained solely by impairments in ToM, rather by compromised core language skills (Norbury, 2005; for a review see Gernsbacher & Pripas-Kapit, 2012). Core language in this study refers to the structural aspects of language such as semantics and grammar. However, the potential link between metaphor comprehension and core language skills have not been fully explored.

## Is Core Language Associated with Metaphor Comprehension in ASD?

To understand a metaphor, the shared semantic features or common ground between two different entities (the “topic” and “vehicle”) need to be grasped (Bühler et al., 2018; van Herwegen & Rundblad, 2018). For example, in the metaphor “Mary is a busy bee”, Mary (the topic—a human being) shares semantic properties (being occupied with work) with a bee (the vehicle—an insect), and this commonality must be grasped to decipher the metaphorical meaning. In addition, metaphors are usually embedded in sentences and advanced command of syntax is necessary to understand them (see Kalandadze et al., 2019).

The few studies that have investigated the association between core language skills and metaphor comprehension in ASD and TD remain inconclusive. Norbury (2005) found that broader semantic knowledge was a significant predictor of metaphor comprehension, but Rundblad and Annaz (2010) argued that this finding was an artefact of the figurative language items included in the measure used. In their own work, Rundblad and Annaz (2010) did not find any significant relationship between core language skills as indexed by receptive vocabulary and metaphor comprehension. This might not be surprising as they only measured word comprehension, which is necessary but not sufficient for metaphor comprehension (Gernsbacher & Pripas-Kapit, 2012). In a recent meta-analysis, Kalandadze et al. (2018) concluded that core language was closely related to figurative language comprehension in ASD. However, since this meta-analysis examined different types of figurative language (e.g., metaphors, idioms, and irony), and understanding each of these may depend on different aspects of core language, more studies on each of these figurative language types are needed. How metaphor comprehension is related to different aspects of core language skills has not been investigated systematically so far. Therefore, we do not know what the extent of difficulties in metaphor comprehension in individuals with ASD compared to those with TD is, and which variables can explain metaphor comprehension difficulties in individuals with ASD.

In this study, we investigated how different aspects of core language contribute to metaphor comprehension in individuals with ASD and TD. We operationalized metaphor comprehension in a within-subjects multi-item experiment, where the literal and metaphorical meaning of words were tested. This allowed us to directly assess the extent to which each participant could understand each metaphor. We expected moderate group-differences in metaphor comprehension that would be explained by different aspects of core language.

## Methods

### Participant Recruitment

After obtaining ethical approval from the Norwegian Ethics committee, we recruited participants from across the country to obtain as large a sample as possible. Participation was voluntary, and the parents or legal guardians of all participants provided informed written consent. Verbal agreement was also obtained from all participants prior to each test session. Invitations to participate in the study were disseminated through the university web page, autism associations, educational psychological services, and schools throughout the country. Control participants were deemed to have TD based on parental reports and nonverbal mental age tests used in this study. They were recruited from schools in the Eastern part of Norway.

Inclusion criteria for participants were an ASD diagnosis (consistent with the ICD-10 criteria; World Health Organization, WHO, 1992), being verbally fluent meaning to have the ability to speak in sentences, and to understand the test instructions. To eliminate the potential impact of bi- or multilingualism, at least one parent of each participant in both groups had to be a native speaker of Norwegian. In addition, the primary language spoken at home had to be Norwegian. No exclusion criteria were applied in terms of comorbidities/co-occurring conditions.

### Sample

A total of 29 children and adolescents with ASD and 31 children and adolescents with TD were recruited for the study. One individual with ASD had to be excluded because the tasks were too difficult for this participant. The final sample consisted of 28 individuals with ASD (three females and 25 males; mean age 146 months (SD = 23 months) and 31 individuals with TD (22 females and nine males; mean age 152 months (SD = 19 months) (see Table 1 for demographic information). Unfortunately, despite our efforts, a gender balance between the groups could not be reached.

**Table 1:** Descriptive statistics for the groups

Measure	ASD (*n* = 28)		TD (*n* = 31)		*∆* (ASD-TD)	*p*	*D*
	*M* (*SD*)	Missing cases	*M* (*SD*)	Missing cases			
CCC-2	68 (15)	0	60 (06)	5	7.33	.019	.66
SRS	149 (15)	0	114 (06)	5	34.92	< .001	3.17
Age in months	146 (23)	0	152 (19)	0	− 5.95	.266	− .28
Nonverbal fluid intelligence	24 (04)	2	24 (04)	0	.07	.946	.02
Core language							
Abstract semantic reasoning	21 (11)	2	26 (07)	0	− 4.17	.074	− .48
Expressive vocabulary	28 (12)	2	36 (08)	0	− 8.02	.004	− .80
Receptive vocabulary	109 (18)	0	117 (10)	0	− 7.57	.049	− .53
Receptive syntax	16 (03)	0	17 (02)	0	− .81	.202	− .35
Metaphor task							
Literal score	20 (06)	0	23 (02)	0	− 2.66	.010	− .65
Metaphor score	15 (09)	0	18 (07)	0	− 3.58	.091	− .46
Difference score	6 (09)	0	5 (08)	0	.93	.672	.11
Total score	35 (11)	0	41 (07)	0	− 6.24	.015	− .67

The *p*-value determines whether the null hypothesis (that there is no mean difference between the two groups) should be rejected. Reshuffling of group labels among participants in each permutation resample included participants with missing values on the measure. Cohen’s *d* standardized group mean difference is reported as the effect size

#### Validation of ASD Diagnosis

The parents or guardians were asked to complete the Norwegian version of the Children’s Communication Checklist (CCC-2) (Bishop, 2003a, 2003b) and the Social Responsiveness Scale (SRS) (Constantino & Gruber, 2012). Both checklists are commonly used to assess pragmatic and social communication skills and repetitive/stereotypical behaviours. The CCC-2 assesses the children's communication in everyday situations and is currently the most psychometrically sound and validated instrument for identifying atypical pragmatic development (Norbury, 2014). The SRS measured autistic symptoms with a higher score reflecting a higher level of autistic behaviours. Both the CCC-2 and SRS can distinguish individuals with ASD from individuals with TD (Bishop, 2003a, 2003b; Constantino & Gruber, 2012).

#### Physical and Mental Age

Physical age was measured in months and ranged from 120 to 196 months in the ASD group and from 120 to 199 months in the TD group. Mental age was measured using the Matrix subtest from the Wechsler Intelligence Scale for Children (WISC-IV) (Wechsler, 2003). Matrix reasoning tasks are suitable for individuals with ASD who might have language and/or motor problems (Boucher, 2017). The participants are shown incomplete visual patterns, each of which has a missing element and are asked to select the missing piece from five options. The number of correct scores was analyzed. The overall average internal consistency reliability of this subtest is .89 and it has been validated for individuals with ASD (Wechsler, 2003).

### Examining Metaphor Comprehension

Because there was no Norwegian *validated or *standardized metaphor comprehension task, we created a multiple-choice task to assess metaphor comprehension. This multiple-choice format reduced the potential confounding impact of verbal communication demands such as meta-linguistic and expressive language skills (Kalandadze et al., 2019 for a comprehensive review of metaphor task properties; Pouscoulous, 2011).

The task included 24 metaphorical items and 24 literal items. Some metaphorical items in the test were translated from existing items in English, while other items were created by the first author in collaboration with a linguist and the last author. The same words used in metaphorical items (e.g., bear = a physically strong and large human being) were used in the literal items (e.g., bear = an animal), so each literal item had a metaphorical counterpart and vice versa.

All 48 items had the same syntactic structure (X = Y). Both the metaphorical and literal expressions were embedded in a short passage of two sentences to aid comprehension. Below each metaphor were multiple-choice responses describing either (A) the intended metaphorical interpretation, (B) the literal interpretation, or (C) an unrelated interpretation (filler/distractor). Below each literal expression were multiple-choice responses describing either (A) the correct literal interpretation, (B) an incorrect literal interpretation, or (C) an incorrect unrelated interpretation. Task development is described in detail in Appendix 1. Six example items (three metaphorical and three literal items) translated from Norwegian into English are presented in Appendix 2.

### Examining Core Language

A multi-measure approach was used to measure different aspects of core language: receptive and expressive vocabulary, abstract semantic reasoning, and receptive syntax.

#### Receptive Vocabulary

Receptive vocabulary was assessed with the Norwegian version of the British Picture Vocabulary Scale (BPVS) (2nd edition; Dunn et al., 1997). After hearing a word, the participants indicated its meaning by selecting a picture from four alternatives. The overall average internal consistency reliability of the entire Norwegian version of the test ***is ***.98 in the norm sample (Lyster et al., 2010). Theoretical BPVS scores range from 0 to 144, with higher scores indicating advanced receptive vocabulary. The raw number of correct scores across items was analyzed.

#### Expressive Vocabulary

The vocabulary subtest of the Wechsler Intelligence Scale for children (WISC-IV; Wechsler, 2003) was used to measure expressive language ability and in-depth vocabulary. In this task, the examiner reads aloud words of increasing complexity and asks the participant to define the meanings of these word. The overall average internal consistency reliability is .89 and the tool is reliable for assessing individuals with ASD (.96) (Wechsler, 2003). Theoretical scores range from 0 to 68, with higher scores indicating advanced expressive vocabulary. The raw number of correct score across items was analyzed.

#### Abstract Semantic Reasoning

Abstract semantic reasoning was assessed with the similarities subtest of the WISC–IV (Wechsler, 2003). The participants are presented with two words that represent common objects or concepts and is asked to describe how they are similar. The overall average internal consistency reliability is .86, and this subtest is a reliable tool for assessing individuals with ASD (.97) (Wechsler, 2003). Theoretical scores range from 0 to 56 with higher scores indicating advanced abstract semantic reasoning. The raw number of correct scores across items was analyzed.

#### Receptive Syntax

Receptive syntax was assessed with the Norwegian version of the Test for Reception of Grammar (TROG-2) (Bishop, 2003a, 2003b; Lyster & Horn, 2009). This test assesses sentence comprehension with a picture-pointing paradigm and multiple-choice response format. Individuals hear a series of sentences and are asked to select a picture from among four alternatives. The test contains 80 stimulus items arranged in blocks of four items per block, which test 20 grammatical contrasts such as prepositions, pronouns, and relative clauses. All items in a block need to be correct to score one point on that block and theoretical scores range from 0 to 20. The blocks increase in grammatical difficulty, and higher scores indicate advanced syntactic knowledge. The overall average internal consistency reliability of the original (English) version is .88 (Bishop, 2003a, 2003b). The raw number of correct score across blocks was analyzed.

#### Procedures

Standard testing procedures, as recommended in the respective manuals, were followed. The procedures were kept as similar as possible for all participants. For all tests, except for the nonverbal fluid intelligence test, the test items were read aloud to the participants. The participants were asked to respond verbally or by pointing to pictures depending on the task. The test took place in a quiet room at the research laboratory, participant's home, or school depending on their preferences. The parents, legal guardians, and/or school staff were invited to attend the testing sessions. To keep the participants motivated and willing to perform the tasks, they were told they could take as many breaks as needed and were asked whether they needed a break during the testing sessions. All testing occurred on the same day.

Before the metaphor comprehension test, a practice item was provided and feedback was given. None of the participants had previously performed the metaphor task. The order of response options for the metaphorical and literal items was randomized for each participant.

The examiners were suitably trained to conduct the tests. Twenty percent of the test data were double-coded by a trained research assistant. The interrater reliability was as follows: receptive syntax (100%), receptive vocabulary (100%), abstract semantic reasoning (100%), expressive vocabulary (100%), mental age (100%), and metaphor task (95.83%).

#### Statistical Analysis

We computed group-wise descriptives for each measure and presented these as mean (M) and standard deviation (SD). For each measure, the difference (Δ) between the ASD group and the TD group was tested using a randomization test approach (Edgington & Onghena, 2007; Pitman, 1937). Then total scores and subscores for the literal and metaphorical items were compared using the same randomization test approach. Randomization-based techniques are optimal for small data sets, can be used to analyze non-random samples, are completely data-dependent, are free of distributional assumptions, and yield exact probability values (Berry et al., 2016). Effect size was measured with Cohen’s *d* (Cohen, 1977).

Next, a random-item random-person explanatory item response modelling approach (De Boeck & Wilson, 2004) was used to relate task performance to individual characteristics such as mental and physical age, group (ASD or TD), and item characteristics. Assessing total scores and then item responses provides a finer perspective of metaphor task performance and accounts for the systematic design of the items. Each item has a literal and a metaphorical variant, allowing a within-subject “metaphor effect” to be determined. Item type is included in the model as a random regression slope to reflect differences in metaphor and literal comprehension between individuals.

The items response models also incorporates the different core language variables to determine their effect on task performance. Results are presented as variance components and logistic regression coefficients and effect sizes as odds ratios. All statistical analyses were performed using R software (R Core Team, 2018). The reliability was calculated using SPSS (version 25.0.0.1; IBM, 2017).

## Results

### Participant Characteristics and Scores Between Groups

Differences between the ASD group and the TD group in the key measures are reported in Table 1 together with the Cohen’s *d* effect size, a standardized group mean difference. The groups did not significantly differ in physical age in months (Δ = − 5.95, *p* = .266, *d* = − .28) or mental age (fluid intelligence score Δ = .07, *p* = .946, *d* = .02). There were significant differences between groups in the CCC-2 scores (Δ = 7.33, *p* = .020, *d* = .66) and the SRS scores (Δ = 34.92, *p* < .001, *d* = 3.17), validating the ASD diagnosis in our sample. The ASD group had lower scores than the TD group in all aspects of core language: expressive vocabulary: Δ = − 8.02, *p* = .004, *d* = − .80; receptive vocabulary: Δ = − 7.57, *p* =. 049, *d* = − .53; abstract semantic reasoning: Δ = − 4.17, *p* = .074, *d* = − .48; and receptive syntax: Δ = − .81, *p* = .202, *d* = − .35, although these differences were only significant for expressive and receptive vocabulary.

The ASD group had significantly lower and more variable scores in the metaphor task than the TD group did (Δ = − 3.58, *p* = .091, *d* = − .46), with the TD group frequently scoring maximum points (i.e., 24) in the literal task items. The participants with ASD got six more literal items correct than metaphorical items and the participants in the TD group got five more literal items correct than metaphorical items. Hence, the TD group outperformed the ASD group overall, but not because of more advanced performance in the metaphor tasks.

### Metaphor Comprehension at the Item Level

For explanatory item response modelling, we removed the data from two participants with ASD because the mental age and core language measures were missing. A total of *n* = 2,832 item responses were given by the remaining 59 participants on the 48 items. All participants answered all items, resulting in 27 unique response patterns and an overall correct response rate of 79%.

#### Model 1

The first baseline model considered that a more able participant will be more likely to give a correct response than a less able participant, regardless of the item, and that a more difficult item is more likely to be answered incorrectly than an easier item is, regardless of the participant. Individual differences among the participants accounted for about 39% (${\sigma }_{person}^{2}=2.77$) of the item response variation, and differences between items accounted for 14% (${\sigma }_{item}^{2}=1.00$) of the item response variation (Table 2). This implies that knowing which participant is responding is more important for predicting the outcome on a particular item than knowing which item is being responded to.

**Table 2:** Explanatory item response modelling of the metaphor task

Model	1		2		3	
Random effects	σ^2^		σ^2^	*r*	σ^2^	*r*
Person	2.77					
Literal baseline			3.78		2.97	
Metaphor-literal gap			7.76	− .66	7.73	− .68
Item	1.00		.09		.09	

Fixed effects	β (SE)	*p*	β (SE)	*p*	β (SE)	*p*
Intercept	2.11 (.28)	< .001	3.46 (.36)	< .001	3.95 (.39)	< .001
Item type			− 2.02 (.47)	< .001	− 1.94 (.46)	< .001
Mental age			.17 (.06)	.011	.17 (.05)	.001
Physical age			.02 (.01)	.030	.02 (.01)	.136
Diagnosis					− 1.25 (.46)	.002
Df	2733		2728		2727	
AIC	2137		1806		2040	
Δχ^2^(df)			341 (5)	< .001	9 (1)	.003

Item type is dummy coded, indicating the metaphorical item variant and with the literal variant as the reference category. Diagnosis is dummy coded, indicating ASD and TD as the reference category. The metaphor-literal gap is the random slope across individuals for the item type effect. The likelihood ratio test statistic Δχ^2^(df) compares each model to its less complex predecessor. *Df* degrees of freedom, *SE* standard error, *AIC* Akaike’s Information Criterion

#### Model 2

The second model incorporated covariate information on persons and items to further improve the item response model (Δχ^2^(5) = 341.46, *p* < 0.001). Instead of assuming a person’s ability to do the whole metaphor task, we distinguished between a personal literal baseline ability and an additional personal penalty that comes into play when solving the metaphorical items. The average odds of answering a metaphorical item correctly was about 7.5 times lower than the odds of answering the corresponding literal item correctly (β = − 2.02 (0.47), *p* < 0.001). This metaphor-literal gap varied highly among participants (${\sigma }_{Type}^{2}=7.76$) and correlated negatively (*r* = − 0.66) with individual differences in literal baseline ability (${\sigma }_{person}^{2}=3.78$). Differentiating between literal and metaphorical items reduced the response variation due to systematic item differences by about 98% (${\sigma }_{item}^{2}=.09$).

Mental and physical age differences accounted for about 20% of the systematic inter-individual differences in response variation (i.e., 23% for literal baseline ability, 12% for the metaphor-literal gap). Participants whose nonverbal intelligence scores were 4 points higher than a participant of similar age had double the odds (exp(4β) = 1.94; β = .17 (.05), *p* = .003) of responding correctly to an item. Participants were 1.29-times more likely (β = .02 (.01), *p* = .049) to respond correctly to an item than a participant with similar nonverbal intelligence who was one year younger.

#### Model 3

Participants with ASD had 3.5-times lower odds (β = − 1.25 (.40), *p* = .002) of responding correctly to an item than participants with TD of matched mental and physical age. No interaction effect between item type and diagnosis was found (Δχ^2^(1) = .40, *p* = .526). In addition, an ASD diagnosis accounted for 10% of systematic inter-individual differences (i.e., 14% for the literal baseline ability, 6% for the metaphor-literal gap component) in metaphor task performance (Δχ^2^(1) = 8.63, *p* = .003). Importantly, about two thirds of the systematic individual differences cannot be explained because of unknown sources of variation between the participants.

### Core Language Aspects and Metaphor Comprehension at the Item Level

Differences in each of the core language variables accounted for an additional 4 to 21% of systematic individual differences in the baseline literal items performance, and for 2 to 9% of systematic individual differences in the metaphor-literal gap. The relationships between expressive vocabulary and overall performance was statistically significant (β = .07 (.02), *p* = .002), and individual differences in expressive vocabulary explained a large part of the observed differences between individuals with ASD and TD (with expressive vocabulary: β = − .70 (.41), *p* = .089 vs without any core language aspects: Table 2, Model 3: β = − 1.25 (.46), *p* = .002). Individual differences in receptive vocabulary and abstract semantic reasoning both were related to the differences in overall performance in core language ability (β = .04 (.02), *p* = .028; and β = .06 (.03), *p* = .047), but the between-group difference was still significant. The inclusion of either expressive vocabulary or abstract semantic reasoning accounted for the differences in task performance attributed to mental age differences. In contrast, no statistically significant support was found for a relation between receptive grammar and metaphor task performance (β = .14 (.09), *p* = .101). Individuals scoring one SD higher on the core language aspect measures are also expected to have higher odds of giving a correct response (1.80, 1.47, 1.46, and 1.24 higher for expressive vocabulary, abstract semantic reasoning, receptive vocabulary, and receptive grammar; Table 3).

**Table 3:** Explanatory item response modelling of the metaphor task in relation to core language ability

SLA	Receptive vocabulary		Expressive vocabulary		Abstract semantic reasoning		Receptive grammar	
Random effects	σ^2^	*R*	σ^2^	*r*	σ^2^	*r*	σ^2^	*r*
Person								
Literal baseline	3.08		2.44		2.97		2.90	
Metaphor-literal gap	7.72	− .72	7.62	− .70	7.89	− .71	7.65	− .69
Item	.09		.09		.09		.09	

Fixed effects	β (SE)	*p*	β (SE)	*p*	β (SE)	*p*	β (SE)	*p*
Intercept	3.84 (.39)	< .001	3.66 (.36)	< .001	3.87 (.38)	< .001	3.88 (.38)	< .001
Item type	− 1.97 (.46)	< .001	− 1.89 (.45)	< .001	− 1.95 (.46)	< .001	− 1.93 (.46)	< .001
Mental age	.12 (.06)	.025	.09 (.05)	.077	.11 (.06)	.063	.09 (.05)	.077
Physical age	.01 (.01)	.667	.01 (.01)	.646	.01 (.01)	.484	.01 (.01)	.646
Diagnosis	− .95 (.41)	.022	− .70 (.41)	.09	− 1.04 (.41)	.011	− 1.11 (.41)	.006
CLS	.04 (.02)	.028	.07 (.02)	.002	.06 (.03)	.047	.14 (.05)	.101
df	2726		2726		2726		2726	
AIC	1796		1792		1797		1798	
Δχ^2^(df)	5 (1)	.030	9 (1)	.002	4 (1)	.048	3 (1)	.101

Coding of variables is similar to those presented in Table 2. *CLS* Core language skills specific core language skills. Receptive vocabulary was measured by the British Picture Vocabulary Scale (BPVS), expressive vocabulary by the Wechsler Intelligence Scale for children (WISC-IV) vocabulary subtest, abstract semantic reasoning by the WISC-IV similarities subtest, and receptive grammar by Test for Reception of Grammar (TROG.2). The likelihood ratio test statistic Δχ^2^(df) compares each model to model 3

## Discussion

In this study, we compared metaphor comprehension task performance between individuals with ASD and TD, and investigated how different aspects of core language explain this performance. We found moderately lower scores in the group of individuals with ASD than in the group of individuals with TD. However, impaired core language skills explained metaphor comprehension difficulties in individuals with ASD.

### Metaphor Comprehension is not a Hallmark of ASD

In line with previous studies (Kalandadze et al., 2019 for a review), individuals with TD generally showed more advanced metaphor comprehension skills than individuals with ASD did, but this was not true for all participants with ASD. There was a high degree of variation in metaphor comprehension within both groups suggesting that difficulties understanding metaphors can be explained by factors other than the ASD diagnosis. Indeed, poor metaphor comprehension is not specific to ASD, and has been observed in individuals with for example schizophrenia (Rossetti et al., 2018) and Developmental Language Disorder (Bühler et al., 2018). In the latter group, impaired core language has been proposed as an underlying variable of metaphor comprehension difficulties (Bühler et al., 2018). This could also be true in ASD as difficulties in different aspects of core language are common among these individuals (Brynskov et al., 2016; Tager-Flusberg & Joseph, 2003).

### Variable Performance in Literal Items Indicates Difficulties in Core Language Skills in Individuals with ASD

Although individuals with TD outperformed individuals with ASD in literal items too, both groups performed better in literal than in metaphor items, corroborating the view that comprehending metaphors and figurative language is more demanding than comprehending literal language (Levorato & Cacciari, 2002; Noveck et al., 2001). This metaphor-literal gap was greater in participants with ASD, possibly because these individuals find it difficult to identify similarities between semantic features. This difficulty may also be caused by impaired cognitive abilities such as ToM (as suggested by Happé, 1993) or executive functioning. Impaired executive functioning skills are also often seen in ASD (see Hill, 2004 for a review). Executive functions such as the mental flexibility to select the common meaning in words, to switch between literal and metaphoric meaning, and to suppress irrelevant literal interpretation, all contribute to metaphor comprehension (Mashal & Kasirer, 2011).

### Item Characteristics, Age, and ASD do not Fully Explain Differences in Task Performance

Differences in task performance were primarily determined by individual ability. While there was a significant difference in performance between the literal and metaphor items, differences between individual items had less of an impact. The metaphor-literal gap was smaller in those participants who performed stronger in the literal variant, and larger in those who performed weaker in the literal variant. This is in line with the logical expectation that core language skills are a prerequisite for metaphorical understanding (Pouscoulous, 2011).

Differences in mental and physical age and ASD did not account for all the differences in performance; the remaining differences were explained by differences in core language skills. This finding fits well with the previous research showing close relationships between metaphor comprehension and core language (Gernsbacher & Pripas-Kapit, 2012).

### Vocabulary is the Most Prominent Language Variable Related to Metaphor Comprehension

One of the most notable findings of this study was that vocabulary, particularly expressive vocabulary as measured with the vocabulary subtest from the WISC-IV (Wechsler, 2003), is central to metaphor comprehension. This finding agrees with previous research that vocabulary is essential for metaphor comprehension (Nippold, 2016; Pouscoulous, 2011). Expressive language is an advanced skill involving conceptualization, formulation and articulation (Levelt, 1995; Norbury, 2014). These abilities are also important for metaphor comprehension. This suggests that individuals with ASD with more advanced vocabulary skills can comprehend metaphors.

### Abstract Semantic Reasoning is also Important for Metaphor Comprehension

We observed that abstract semantic reasoning, as measured with the similarities subtest of the WISC–IV (Wechsler, 2003) was important for metaphor comprehension. This is not surprising since a person needs to identify the shared properties between two elements (i.e., topic and a vehicle) before they can understand a metaphor (Pouscoulous, 2011). The test we used to measure abstract semantic reasoning also requires to identify the similarities between two words. The test also requires expressive language ability, further indicating that participants who performed well in the metaphor task had advanced linguistic and non-linguistic abilities.

### Receptive Grammar Alone cannot Explain Variation in Metaphor Comprehension

Individuals with TD and ASD both scored well on receptive grammar, and receptive grammar ability did not affect metaphor task performance. This may be because we used a simple X = Y syntactic structure in our task with little syntactic variation between the items. A task with more complex or different syntactic structures might have been more strongly associated with metaphor comprehension. For example, predicate metaphors use a verb to create metaphorical meaning, e.g., “when Taro plays soccer, no one at his school comes close to him” (Adachi et al., 2004). Here, an understanding of the conceptual features of a verb is needed (Chen et al., 2008). Furthermore, the test we used to examine receptive grammar (TROG test) may not be sensitive enough to detect subtle differences between individuals, and the sensitivity may vary between languages the test is translated into. Although TROG was the only sentence comprehension test in Norwegian that was standardised for adolescents up to 16 years of age it may not be sensitive enough for the groups under investigation. Indeed, the manual of the Norwegian translation of the TROG (Lyster & Horn, 2009) states that the scores plateau somewhat after 8–9 years in individuals with TD, with small standard deviations for the oldest participants.

### Limitations

One of the main limitations of this study is the sample size that influenced how many potentially relevant associated variables we could examine. For example, we did not control for ToM, which previously was found to be an explanation of difficulties in metaphor comprehension in ASD (Happé, 1993). Small sample size, in addition to the gender imbalance and the inclusion of only verbally fluent individuals with ASD, may prevent generalization of our findings to all individuals with ASD. However, although our sample size is small in the sense of statistics, the size of our sample is in line with other studies in the field. In particular, the mean sample size in studies on metaphor comprehension included in the recent meta-analysis was 24 (SD = 15.01) (Kalandadze et al., 2019). Also, co-existence of a range of comorbide/co-occuring conditions together with ASD is common (Boucher, 2017). Therefore, including verbally fluent individuals with ASD does not eliminate the chance that comorbid/co-occurring conditions or difficulties in skills that we did not control for could have affected performance on metaphor comprehension task.

Another limitation that should be considered when interpreting the results of this study is that the metaphor task we used was created for this specific study due to the lack of such a measure in Norwegian. The task is therefore not a standardized test. However, several aspects were considered to ensure the quality of the metaphor task (see the supplementary files for details).

Another measurement-related caveat is that, unfortunately, no standardized tests of expressive vocabulary and semantics in Norwegian suitable for our age group existed. Therefore, we used the WISC-IV subtests (Wechsler, 2003) to measure these skills. Our results therefore can also indicate that metaphor comprehension may be closely related to verbal mental age. However, we do not have the sufficient information about the participants’ non-verbal intelligence to draw any conclusions on the potential relationship between the non-verbal intelligence and metaphor comprehension in our sample.

One final aspect that should be mentioned here is that this study was conducted in a controlled setting and not in a naturalistic environment. The demand to interact with the examiner would potentially have influenced the performance. Therefore, these results might not be generalisable to naturalistic contexts. Although we encourage the readers as well as researchers who plan to conduct similar studies to consider these limitations, we would like to emphasize that the current study fills the gap in the literature and contributes to the accumulation of knowledge in the field of metaphor comprehension in ASD.

### Potential Implications for Research and Practice

Our findings highlight the need to consider variability in core language skills when studying metaphor comprehension in individuals with ASD. We showed that Item response theory is a useful analytic approach to use with this respect. The wide variability we observed in performance on the language tasks underlines the need for further research into the internal (e.g., executive functions) and external (e.g., socioeconomic status, exposure to metaphors) factors that might be related to metaphor comprehension.

When possible, future research should include more valid measures of different aspects of core language than we did in this study. Although pure language measures are difficult to find, some language measures make it more possible than others to tease apart language skills from other cognitive abilities.

Our results illustrate the importance of focusing on core language skills in addition to teach individuals with ASD strategies to understand metaphors in educational and clinical settings. Individuals with ASD and individuals with TD should receive educational support targeting their language including metaphoric language that is specifically tailored to their individual needs.

## Conclusions

Although many individuals with ASD find it harder to understand metaphors than individuals with TD do, these difficulties are not a hallmark feature of ASD. Instead, the ability to comprehend metaphors depends on the different aspects of the individual’s core language skills. Future research on metaphor comprehension needs to focus on variability in core language skills among individuals with ASD and TD.

## Supplementary Information

Below is the link to the electronic supplementary material.

Electronic supplementary material 1 (DOCX 31 kb)
