# Supplementary material for: Metaphor Comprehension in Individuals with Autism Spectrum Disorder: Core Language Skills Matter
Source: J Autism Dev Disord. 2021 Mar 10;52(1):316–26. doi: 10.1007/s10803-021-04922-z (PMC8732923; doi:10.1007/s10803-021-04922-z)
Supplement: Supplementary file 1 — Electronic supplementary material 1 (DOCX 31 kb) [file 10803_2021_4922_MOESM1_ESM.docx]

Appendix 1

**The metaphor task development procedure**

Several steps were taken to ensure the reliability and validity of the metaphor comprehension measure. *First*, 70 expressions containing metaphors were either selected from [Removed for review] dictionaries or translated from other languages. *Second*, the selected metaphorical expressions were discussed thoroughly with experts in linguistics and speech and language therapy, as well as in psycholinguistics and ASD. *Third*, the metaphors were then embedded in a two-sentence context to aid comprehension. Several considerations were made in the process of creating the context: a) the context was supportive but not priming; b) the lengths of the sentences was approximately the same; c) sentences did not include difficult or ambiguous words that could confuse the participants. The appropriateness of the context to support metaphor comprehension was also discussed with experts and changes were made as a result. *Fourth*, as an additional step in establishing the comprehensibility of the expressions in the given context, the task was piloted with three adolescents with TD (their responses are not included in the analysis in the study). After the testing session, the metaphorical items were discussed with these adolescents. With this last step we checked the accessibility of the metaphorical meaning for all the items in the passage and ensured that the context provided appropriate clues for interpretation. Only metaphorical items that were evaluated as comprehensible by all 3 pilot participants in the given context were selected for inclusion in the metaphor task.

Discussions regarding the metaphor task with the experts also concerned the number of items to include in order to maintain reliability but at the same time not cause fatigue among the participants. Among the items that were evaluated as appropriate, we selected 24 items.

*Scoring and internal consistency reliability*. The total metaphor raw scores were calculated by summing the scores attributed to each item on a scale of 0-2 (0=incorrect irrelevant alternative or no response; 1=incorrect literal alternative; 2=correct metaphorical answer). The total theoretical score ranged from 0 to 72 (24 x 2) for metaphorical items + (24 x 1) for literal items) with 0 indicating low level of comprehension of metaphorical and literal items, and a score of 72 or 24 indicated high level of comprehension of metaphorical and literal items. The score reliability, indexed by Cronbach alpha, was .959 for metaphor items only (24), and Cronbach alpha was .944 for both metaphorical and literal items together (48).

Appendix 2

| **Metaphorical item** | **Literal item** |
| --- | --- |
| I frequently have conversations with my Norwegian teacher. She is an *encyclopaedia* (metaphor).  1) The [Removed for review] teacher knows everything  2) The [Removed for review] teacher is a reference work  3) The [Removed for review] teacher is young | I can see a thick book on the shelf. It is an *encyclopaedia* (literal).   1. It is a reference work 2. It is a sofa 3. It is an umbrella |
| Peter filled his apartment with furniture. He is a *bear*.  1) Peter is strong  2) Peter is a big animal  3) Peter is a kindergarten teacher | Peter sees something moving in the forest. This is a *bear.*   1. Peter sees a big animal 2. Peter sees a young fireman 3. Peter sees a small animal |
| Anne is looking at herself in the mirror. Her hairdresser is a *magician.*   1. Her hairdresser is employed in the circus 2. Her magician is slow 3. Her magician is doing a good job | The man is entertaining the kids. He is a *magician.*   1. The man is employed in the circus 2. The man is an owner of the house 3. The man is a tenant |
